# Subtotal splenectomy for the treatment of chronic lymphocytic leukemia

**DOI:** 10.1038/bcj.2015.16

**Published:** 2015-03-13

**Authors:** A Petroianu

**Affiliations:** 1 Department of Surgery, Medical School of the Federal University of Minas Gerais, Belo Horizonte, Brazil

In an attempt to maintain at least part of the splenic function when removal of the spleen is indicated, since 1984 we have performed subtotal splenectomy combined with central splenorenal shunt or with portal–variceal disconnection for the treatment of portal hypertension in 129 patients.^1, 2, 3, 4, 5, 6^ Subtotal splenectomy has also been used by us to treat 93 patients with severe splenic trauma,^7, 8^ eighteen patients with myeloid metaplasia,^9^ nine patients with Gaucher's disease,^10^ five patients with retarded growth and sexual development associated with splenomegaly,^11^ three patients with severe splenic pain due to extensive intraparenchymal thrombosis,^12^ one patient with a splenic hemangioma,^13^ one patient with a splenic abscess^14^ and one patient with a cystadenoma of pancreatic tail.^15^

Splenectomy may be indicated in the management of selected patients with chronic lymphocytic leukemia (CLL), but in most of the cases, this procedure is accompanied by a high morbidity and mortality, mainly due to severe sepsis. The postoperative adverse effects of the asplenic condition lead to the exclusion of surgical treatment in most patients with leukemia.

The purpose of the splenectomy is to obtain a better control of refractory leukemic activity, when it does not respond to chemotherapy. The surgical procedure also alleviates the abdominal discomfort motivated by a very large spleen.

As the purpose of splenectomy is not to make the patient asplenic, a partial splenectomy may achieve the objectives of the surgical indications without the adverse side effects of the total removal of the spleen. However, good results are transitory because the spleen regrows in a short period of time due to the intense blood flow through the splenic pedicle.

To avoid the adverse effects of total and partial splenectomies when these procedures are indicated, the present study proposes the subtotal splenectomy, preserving the superior splenic pole supplied only by the splenogastric vessels to control the refractory leukemic activity and the abdominal discomfort provoked by a very large spleen in the presence of CLL.

Five patients of the oncological services of the Hospital of Clinics and of the Santa Casa Hospital of Belo Horizonte city, Brazil were referred to us for surgical treatment of their spleen, due to refractory leukemic activities.

The patients were four men and one woman with ages between 39 and 81 years old. All these patients have been subjected to chemotherapies and presented intense abdominal discomfort due to very large spleens. On the basis of our previous experience with subtotal splenectomy and of the literature that indicates partial splenectomy to control leukemia, a subtotal splenectomy was associated to the oncological treatment.^1, 2, 3, 4, 5, 6, 7, 8, 9, 10, 11, 12, 13, 14, 15^

All the five patients were subjected to the same surgical procedure, as follows. The abdominal cavity was entered through a transverse left laparotomy; the splenic artery was tied in the retrogastric space. The spleen was displaced upward and its ligaments were dissected and divided with an electrocautery (Figure 1a). The hilum and the inferior splenic vessels were tied and cut. Care was taken to preserve the splenogastric vessels that maintain the vitality of the superior splenic pole. The spleen was cut at the level of the limit between the normal color of the superior portion of the organ and the darker colored rest of the spleen. Two wide flaps (anterior and posterior) of the splenic capsule were retained. The bleeding large vessels of parenchyma were sutured with 4-0 chromic catgut. A continuous 3-0 chromic catgut suture was used to close the two flaps of the splenic capsule (Figure 1b). The superior pole was returned to its normal position and then it was sutured to the diaphragm with one stitch using 2-0 chromic catgut. The blood loss was minimal in all cases and no blood transfusion was necessary. The portion of the removed spleen weighed 3950, 4440, 4620, 5200 and 6800 g.

Histopathological analyses of the spleens confirmed the leukemic infiltration. The five patients had uneventful postoperative courses and were discharged from the hospital on the third, fifth (two patients) and sixth (two patients) postoperative days. After the surgical recovery, they continue to be treated by the oncological services.

During the 3- to 8-year follow-up, the patients did not present severe infection and the leukemia was adequately controlled with chemotherapy. The only patient who died was the 81-year-old man, at the age of 89, due to complications of a chronic lung emphysema.

Scintigraphic images of the splenic remnant using 99m-technetium sulfur colloid were positive in all postoperative exams, showing the splenic vitality and the presence of the phagocytic function. According to the computed tomography scan exams, the dimensions of the splenic remnants did not modify during the follow-up periods.

Chemotherapy is the usual treatment of CLL, and most of patients survive many years under drug control. However, the huge growth of the spleen, which may occur in some patients, leads to intense abdominal discomfort and therapy failure. In such cases, total splenectomy has been indicated. This procedure alleviates the symptoms and improves the results of chemotherapy.

It is well known that total splenectomy reduces the immune defenses and may be followed by severe sepsis associated to precocious deaths. The complications of asplenic status are more frequent in previously immune-suppressed patients due to leukemia. Chemotherapy worsens the organic defenses and enhances the mortality.

As the removal of the spleen is not necessary to treat CLL, partial splenectomy was proposed to reduce the influence of the splenic growth on the symptoms and on the hematologic system. Previous experiences with partial splenectomy to treat very large spleens due to myeloid metaplasia or Gaucher's disease have shown the regrowth of the splenic remnant when the vascular pedicle is preserved. Then, after a transitory improvement of the clinical aspects during the early postoperative period, the symptoms and the hematological disorders return, and sometimes a new surgical procedure is indicated to remove the rest of the spleen.

Subtotal splenectomy preserving the superior splenic pole supplied only by the splenogastric vessels has shown that these vessels are not able to support a larger amount of spleen than the superior pole. This procedure has the advantage of preserving part of the spleen without the risk of its regrowth and recurrence of symptoms.^5^ Further studies are needed to confirm the advantages of this procedure.

In conclusion, subtotal splenectomy is efficacious to preserve the splenic functions and to prevent the adverse effects of a large spleen on the treatment of chronic lymphocytic leukemia.

## Figures and Tables

**Figure 1:**
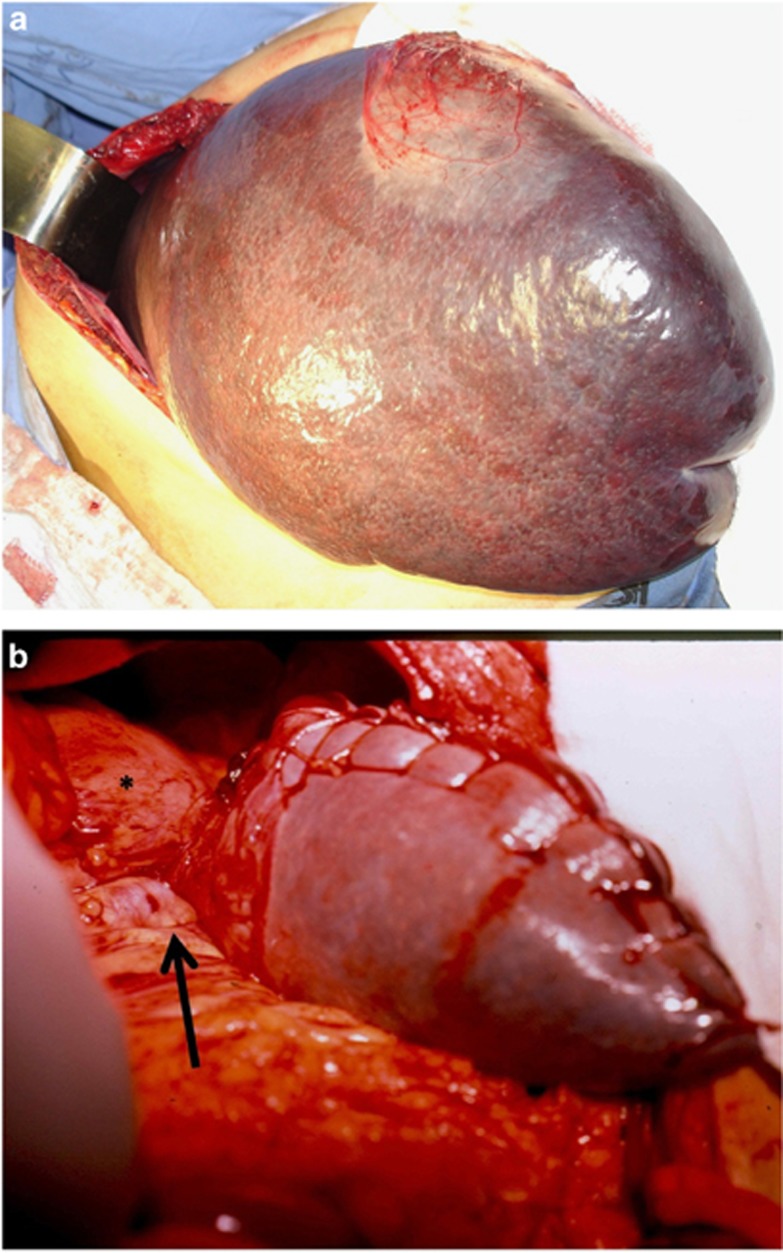
Surgical views of subtotal splenectomy for treatment of a patient with CLL. (**a**) The very large spleen mobilized to the surgical field after the section of the splenic ligaments. (**b**) The superior splenic pole after subtotal splenectomy. Observe the splenogastric vessels (arrow) between the stomach (*) and the splenic remnant.
